# Activation of AMP-Activated Protein Kinase by 3,3′-Diindolylmethane (DIM) Is Associated with Human Prostate Cancer Cell Death *In Vitro* and *In Vivo*

**DOI:** 10.1371/journal.pone.0047186

**Published:** 2012-10-09

**Authors:** Di Chen, Sanjeev Banerjee, Qiuzhi C. Cui, Dejuan Kong, Fazlul H. Sarkar, Q. Ping Dou

**Affiliations:** 1 Department of Oncology, Barbara Ann Karmanos Cancer Institute, Wayne State University, School of Medicine, Detroit, Michigan, United States of America; 2 Department of Pathology, Barbara Ann Karmanos Cancer Institute, Wayne State University, School of Medicine, Detroit, Michigan, United States of America; Sun Yat-sen University Cancer Center, China

## Abstract

There is a large body of scientific evidence suggesting that 3,3′-Diindolylmethane (DIM), a compound derived from the digestion of indole-3-carbinol, which is abundant in cruciferous vegetables, harbors anti-tumor activity *in vitro* and *in vivo*. Accumulating evidence suggests that AMP-activated protein kinase (AMPK) plays an essential role in cellular energy homeostasis and tumor development and that targeting AMPK may be a promising therapeutic option for cancer treatment in the clinic. We previously reported that a formulated DIM (BR-DIM; hereafter referred as B-DIM) with higher bioavailability was able to induce apoptosis and inhibit cell growth, angiogenesis, and invasion of prostate cancer cells. However, the precise molecular mechanism(s) for the anti-cancer effects of B-DIM have not been fully elucidated. In the present study, we investigated whether AMP-activated protein kinase (AMPK) is a molecular target of B-DIM in human prostate cancer cells. Our results showed, for the first time, that B-DIM could activate the AMPK signaling pathway, associated with suppression of the mammalian target of rapamycin (mTOR), down-regulation of androgen receptor (AR) expression, and induction of apoptosis in both androgen-sensitive LNCaP and androgen-insensitive C4-2B prostate cancer cells. B-DIM also activates AMPK and down-regulates AR in androgen-independent C4-2B prostate tumor xenografts in SCID mice. These results suggest that B-DIM could be used as a potential anti-cancer agent in the clinic for prevention and/or treatment of prostate cancer regardless of androgen responsiveness, although functional AR may be required.

## Introduction

AMP-activated protein kinase (AMPK) is expressed in all eukaryotic cells and is a critical enzyme that plays an essential role in cellular energy homeostasis, as well as controlling processes related to tumor development including cell cycle progression, cell proliferation, protein synthesis, and survival. Therefore, as an anti-cancer target, AMPK has received intensive attention in recent years. Mammalian AMPK is a trimeric serine/threonine protein kinase composed of a catalytic α subunit and two regulatory subunits, β and γ. AMPK is activated through phosphorylation of Thr-172 on the α subunit by an energy-depleting stress, such as increased ratios of AMP/ATP [1] and ADP/ATP [2], or stimulated by cellular kinases including liver kinase B1 (LKB1) [3]–[4] and calmodulin-dependent protein kinase kinase (CaMKK) [5]. Once activated, AMPK plays two major functions, metabolic and non-metabolic. In the regulation of metabolic process, AMPK phosphorylates serine moieties in many target proteins and results in switching on of catabolic pathways to activate ATP-generating processes including the uptake and oxidation of glucose and fatty acids, and switching off of anabolic pathways including protein, fatty acid and cholesterol syntheses, which consume ATP [6]. Regarding non-metabolic functions of AMPK, activation of AMPK can induce cell cycle arrest and inhibit cell proliferation and protein synthesis in malignant cells through multiple mechanisms such as the accumulation of tumor suppressor factor p53 and the cyclin-dependent kinase inhibitors p21 and p27 [7], as well as down-regulation of the mTOR pathway [8]–[9]. Extensive research supports the role of AMPK in cancer prevention and therapeutics, suggesting that targeting AMPK may be a promising option for cancer treatment.

To that end, metformin, an anti-diabetic drug, has been shown to activate AMPK, raising a hypothesis that metformin may reduce the risk of cancer in patients with type 2 diabetes through activation of the AMPK pathway [10]. Indeed, reports from clinical studies have demonstrated that diabetic patients treated with metformin had a significantly lower rate of cancer incidences and cancer-related mortality compared with patients exposed to other anti-diabetic medicines [10]–[12]. Pre-clinical studies have also shown that metformin not only inhibits growth of cultured cancer cells [13]–[14] and tumors in mice [15], but also selectively targets cancer stem cells [16].

Besides metformin, some natural compounds, including quercetin, genistein [17], capsaicin, EGCG [18], and curcumin [19], have been shown to have anticancer effects associated with activation of the AMPK signaling pathway. In fact, natural products have been the most productive source of leads for the development of anti-cancer drugs. According to the literature, approximately 73% of anticancer drugs were discovered from natural origins or derived from natural compounds over the past half a century [20]. The natural compound indole-3-carbinol (I3C), which is found at relatively high levels in cruciferous vegetables such as broccoli and cabbage, and its dimer 3,3′-diindolylmethane (DIM) have shown anti-tumor activity *in vitro* and *in vivo* [21]–[22]. Recently, we reported that a formulated DIM (BR-DIM, obtained from BioResponse Nutrients, LLC., Boulder, Colorado, hereafter abbreviated as B-DIM), showed approximately 50% higher bioavailability *in vivo* [23] compared with DIM. B-DIM induced apoptosis and inhibited cell growth, angiogenesis, and invasion of prostate cancer cells, which is associated with regulation of Akt, NF-κB, VEGF and AR signaling pathways [24]–[25]. In addition, recent results have shown that B-DIM treatment of prostate cancer cells *in vitro* or B-DIM intervention in patients with prostate cancer led to the nuclear exclusion of AR associated with activation of miR-34a [24]. However, the precise molecular mechanism(s) by which B-DIM plays its anti-cancer and cancer-preventive roles have not been fully elucidated; more specifically, it has not been reported whether the biological activity of B-DIM is related to induction of AMPK signaling.

Therefore, in the current study, we investigated the effects of B-DIM on AMPK signaling and its related downstream targets in both androgen-sensitive LNCaP and androgen-insensitive C4-2B prostate cancer cells containing functional AR. Our results showed, for the first time, that B-DIM could function as an AMPK activator. Activation of AMPK by B-DIM resulted in the down-regulation of AR and prostate-specific antigen (PSA) expression, and caused induction of cell apoptosis, suppression of mTOR pathway, and inhibition of prostasphere formation in human prostate cancer cells *in vitro* and *in vivo*. Our findings also demonstrated that the AMPK pathway is one of the novel molecular targets of B-DIM for its anti-cancer effects against human prostate cancer.

## Methods

### Cell Culture, Protein Extraction, and Western Blot Assay

Human prostate cancer C4-2B (obtained from Professor Leland Chung, Emory University, School of Medicine, Atlanta, GA; and currently at Cedars-Sinai, Los Angeles, CA) and LNCaP (American Type Culture Collection Manassas, VA, USA) cells were grown in RPMI 1640 medium (Invitrogen, Carlsbad, CA) supplemented with 10% fetal calf serum (FCS), 100 µg/ml streptomycin, 100 units/ml penicillin, and 2 mM glutamine, in a humidified incubator with 5% CO_2_ and 95% air at 37°C. A whole cell extract was prepared as previously described [26]. For Western blot analysis, an equal amount of protein from each cell extract was subjected to denaturing polyacrylamide gel electrophoresis (SDS-PAGE) and transferred to nitrocellulose membranes. Individual membranes were probed with indicated antibodies. Immunoreactive bands were developed using horseradish peroxidase conjugated secondary antibodies and SuperSignal WestPico chemiluminescent substrate (Pierce) and visualized using X-ray film.

### Prostasphere Formation Assay

Prostasphere formation assay was performed to assess the capacity of cancer stem cell self-renewal following our published procedure [27]. Briefly, single cell suspensions of C4-2B cells were thoroughly suspended and plated in ultra low adherent-wells of 6-well plates (Corning, Lowell, MA) at 1,000 cells/well in 1.5 ml of sphere formation medium (1∶1 DMEM/F12 medium supplemented with 50 units/ml penicillin, 50 mg/ml streptomycin, B-27, and N-2). One milliliter of sphere formation medium was added every 3 days. After 6 days of incubation with different concentrations of B-DIM or metformin (as a control), the formed spheres were collected by centrifugation at 300×g for 5 minutes and prostasphere numbers were counted under an inverted phase-contrast microscope. The proportion of sphere-generating cells was calculated by dividing the number of cells seeded by the number of prostaspheres.

### Experimental Animals

Male homozygous CB-17 SCID mice (4–5 weeks old) were purchased from Taconic Farms (Germantown, NY). The mice were housed and maintained under sterile conditions in facilities accredited by the American Association for the Accreditation of Laboratory Animal Care and in accordance with current regulations and standards of the U.S. Department of Agriculture, U.S. Department of Health and Human Services, and NIH. The mice were used in accordance with Animal Care and Use Guidelines of Wayne State University under a protocol approved by the Wayne State University Animal Care and Use Committee. Mice received Lab Diet 5021 (Purina Mills, Inc., Richmond, IN).

### Human Bone and Implantation of Tumor Cells

Human male fetal bone tissue was obtained by a third party nonprofit organization (Advanced Bioscience Resources, Alameda, CA), and written informed consent was obtained from the donor family, consistent with regulations issued by each state involved and the federal government. After one week of acclimatization, the mice were implanted with a single human fetal bone fragment as described previously [28]–[29]. C4-2B cells were harvested from subconfluent cultures after a brief exposure to 0.25% trypsin and 0.2% EDTA. Trypsinization was stopped by adding a medium containing 10% FBS. The cells were washed once in serum-free medium and resuspended. Only suspensions consisting of a single cell with >90% viability were used for the injections. Cells (1×10^6^) in 20-µL serum-free RPMI medium were injected intraosseously by insertion of a 27-gauge needle and Hamilton syringe through the mouse skin directly into the marrow surface of the previously implanted bone. In our previous experience with this model, we found a tumor uptake rate of >95% compared to skin xenograft wherein the tumor uptake rate was comparatively less with prolonged latency period. As soon as the majority of the bone implants began to enlarge (now called a “bone tumor”) as determined by caliper measurements (30th day after cancer cell injection), mice were randomized into the following treatment groups (n = 7): (a) untreated control; (b) only B-DIM, 5 mg/mice fed everyday orally by gavage for 4 weeks since the initiation of therapy. The volume of the bone tumor in each group was determined by twice weekly caliper measurements. The body weight of mice in each group was also measured. All mice were euthanized one day after the last dose of B-DIM treatment (5 weeks) because large tumors were formed in the control mice, which required termination, and their final body weight and tumor volume were recorded. On autopsy, the tumor was neatly excised, freed of any extraneous adhering tissue, and part of the tissue was fixed in formalin and embedded in paraffin for immunostaining and H&E staining for confirming the presence of tumor.

## Results

### B-DIM Activates AMPK Signaling in Human Prostate Cancer Cells

**Figure 1:**
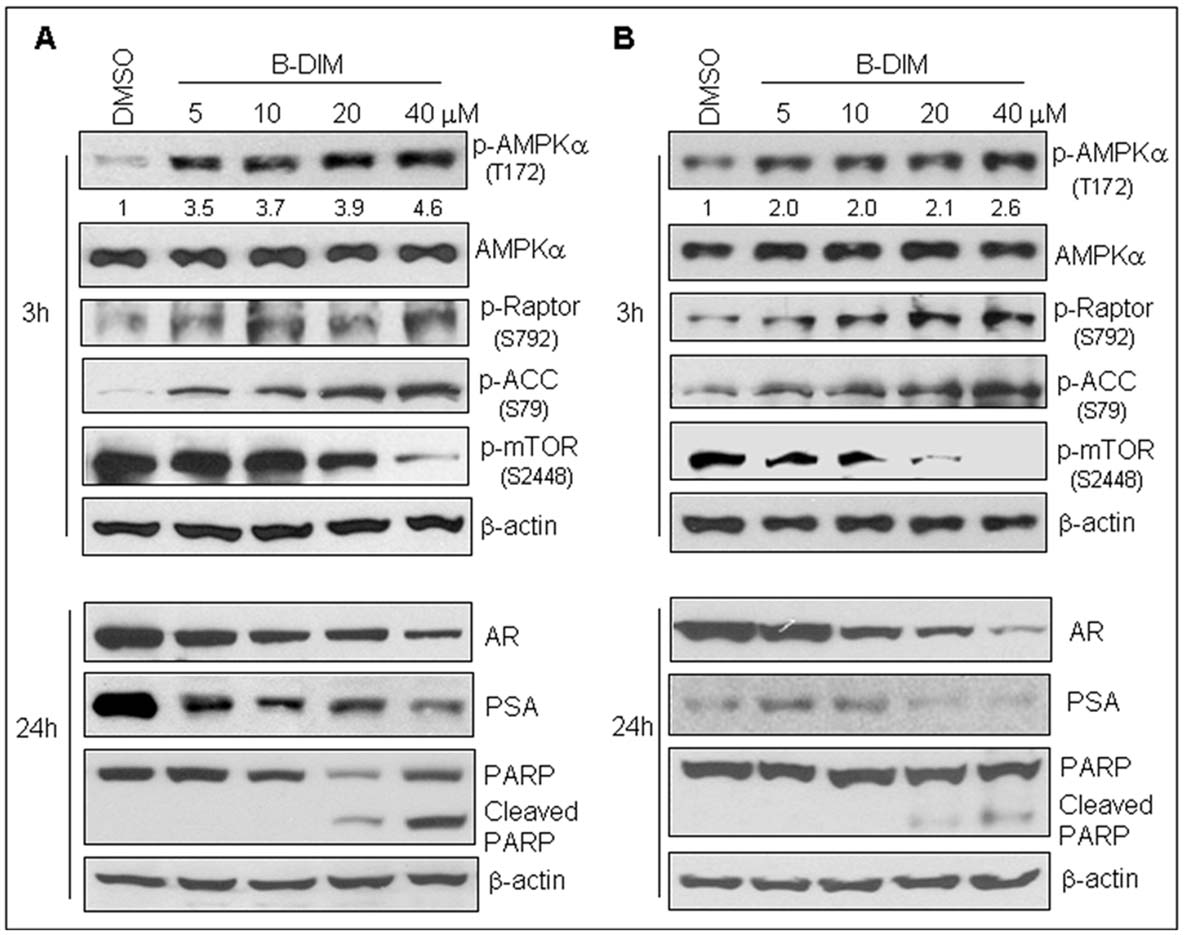
B-DIM activates the AMPK pathway, resulting in inhibition of AR and PSA expression and induction of apoptosis in prostate cancer cells. Human prostate cancer C4-2B (A) or LNCaP (B) cells were treated with indicated concentrations of B-DIM for 3 hours to measure protein levels of phosphor-AMPKα, AMPKα, phosphor-Raptor, phosphor-ACC, phosphor-mTOR, or for 24 hours to measure protein levels of AR, PSA or PARP cleavage by Western blot analysis. Measurement of β-actin served as loading controls. The numbers underneath the Western results of phosphor-AMPKα indicate quantified normalized phosphor-AMPKα and β-actin ratios.

We assessed whether the AMPK signaling pathway could be one of the molecular targets of B-DIM in both androgen-insensitive C4-2B (Fig. 1A) and androgen-sensitive LNCaP (Fig. 1B) prostate cancer cells. Both cell lines were treated with different concentrations of B-DIM for 3 hours, and cell lysates of the treated cells were analyzed by Western blot to measure the levels of phosphor-AMPKα (T172), an active form of AMPK protein, as well as some downstream target proteins. The results showed that the level phosphor-AMPKα in both prostate cancer cell lines treated with B-DIM increased in a dose-dependent manner (Fig. 1A, B). As a control, the total AMPK protein levels remained relatively unchanged (Fig. 1A, B). Both regulatory-associated protein of mTOR (Raptor) and acetyl-CoA carboxylase (ACC) are direct downstream substrates of AMPK, as activated AMPK is able to phosphorylate Raptor protein on serine residue 792 [8] and ACC protein on serine residue 79 [30]–[31]. Treatment with B-DIM resulted in increased levels of phosphor-Raptor (S792) and phosphor-ACC (S79) in the treated cells (Fig. 1A, B), further supporting that B-DIM is an AMPK activator. mTOR is a downstream signaling pathway of AMPK, and activation of AMPK can inhibit the mTOR signaling pathway [32]. Our data also revealed that activation of AMPK by B-DIM could suppress the mTOR pathway, as measured by decreased protein level of phosphor-mTOR (Fig. 1A, B), demonstrating, for the first time, the functionality of B-DIM as an AMPK activator in both AR-sensitive and AR-insensitive prostate cell lines. The inactivation of mTOR by B-DIM is consistent with our previously published report [33].

### Activation of AMPK by B-DIM at Early Hours is Associated with Subsequent Down-regulation of AR and PSA Protein Expression and Induction of Apoptosis in Human Prostate Cancer Cells

The results displayed above show that B-DIM harbors AMPK-activating property. We then investigated whether activation of AMPK by B-DIM could result in apoptotic cell death and inhibit the expression of prostate cancer signature proteins, such as AR and PSA. We showed previously that activation of AMPK signaling by B-DIM is an early event that occurs as early as three hour-treatment (the upper panel of Fig. 1A, B). We found that further treatment of C4-2B and LNCaP cells with B-DIM for up to 24 hours significantly decreased the expression levels of AR and PSA in a dose-dependent manner, and also resulted in apoptotic cell death as measured by PARP cleavage (the lower panel of Fig. 1A, B). These data suggest that activation of the AMPK signaling pathway is one of the major targets of B-DIM leading to the induction of apoptotic cell death of prostate cancer cells.

### Activation of AMPK by B-DIM can be Blocked by an AMPK Inhibitor

**Figure 2:**
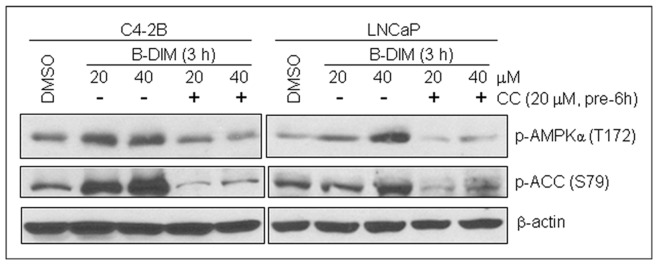
AMPK inhibitor Compound C can block AMPK activation by B-DIM in prostate cancer cells. Prostate cancer C4-2B and LNCaP cells were pre-treated with 20 µM of AMPK inhibitor Compound C (CC) for 6 hours, followed by co-treatment with indicated concentrations of B-DIM for 3 hours. Cell extracts of the treated cells were immunoblotted for anti-phosphor-AMPKα, phosphor-ACC or β-actin antibodies.

Compound C was developed as a selective inhibitor of AMPK [34]. We hypothesized that if AMPK is an essential molecular target of B-DIM, co-treatment with Compound C should attenuate or block the effects of B-DIM on prostate cancer cells. To test this hypothesis, prostate cancer C4-2B and LNCaP cells were pre-treated with either 20 µM of Compound C or DMSO for 6 hours and then co-treated with B-DIM at two concentrations for additional 3 hours. The immunoblotting results showed that in both prostate cancer cell lines treated with B-DIM alone, AMPK signaling was activated as measured by a dose-dependent increase in the phosphorylation of AMPKα (T172) and the phosphorylation of Ser79-ACC (Fig. 2), a direct substrate of AMPK that is widely used as a detector of AMPK activation [35]–[37]. However the protein levels of phosphor-AMPKα and phosphor-ACC were dramatically decreased in cells pre-treated and co-treated with Compound C (Fig. 2), suggesting that B-DIM-activated AMPK can be blocked by an AMPK inhibitor.

### B-DIM Enhances AR Suppressing-effect of Anti-AR Drug Casodex

**Figure 3:**
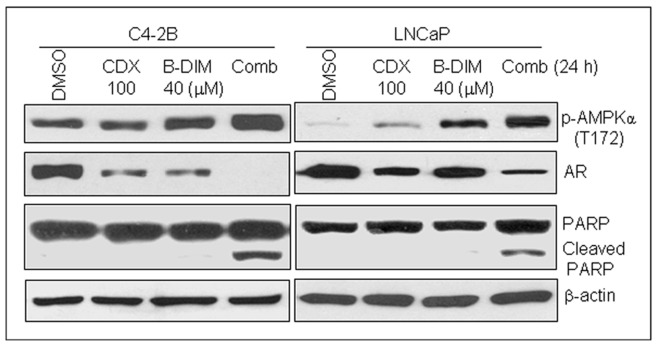
B-DIM enhances the effects of AR inhibitor casodex in prostate cancer cells, associated with increased activation of AMPK. Prostate cancer C4-2B and LNCaP cells were treated with 100 µM of casodex, 40 µM of B-DIM alone or a combination of casodex and B-DIM for 24 hours, followed by Western blot analysis using anti-phosphor-AMPKα, AR, PARP or β-actin antibodies.

One of the current treatment strategies for advanced prostate cancer is to suppress AR function by castration and anti-androgens. Casodex is an anti-androgen drug clinically used for patients with metastatic/advanced stage prostate cancer, and works by binding and preventing the activation of the AR. Our previous studies have shown that B-DIM is able to inhibit AR expression in prostate cancer cells [24]. We further hypothesized that the combination of B-DIM with casodex may have a synergetic effect on the inhibition of AR expression and induction of apoptosis in prostate cancer cells, which may be associated with the activation of AMPK. To test this hypothesis we co-treated both prostate cancer cell lines with 100 µM of casodex and 40 µM of B-DIM for 24 hours, and treatment with each agent alone served as controls. The data show that co-treatment of C4-2B and LNCaP cells with casodex and B-DIM significantly decreased the expression level of AR and increased apoptosis-associated PARP cleavage compared to each treatment alone, and the synergetic or additive effect was accompanied by increased AMPK activation (Fig. 3).

### Both B-DIM and Metformin Significantly Inhibits Prostasphere Formation

**Figure 4:**
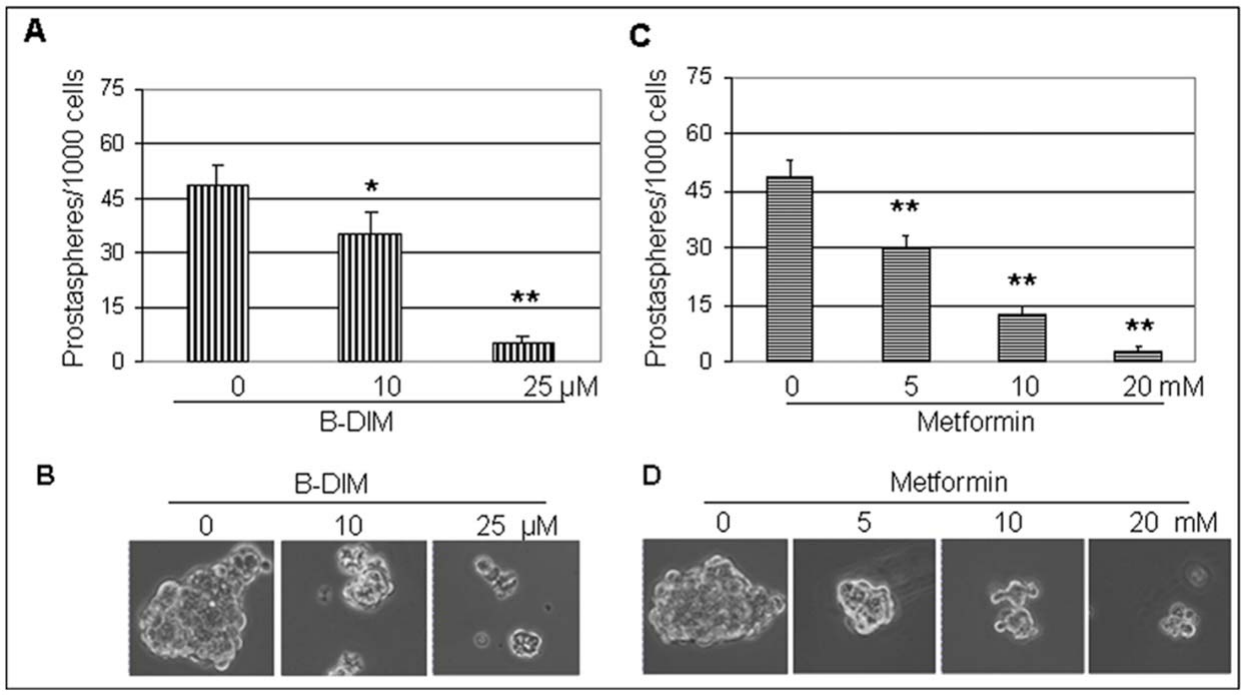
Treatment with B-DIM or metformin inhibits prostasphere-forming ability. C4-2B cells were treated with indicated doses of B-DIM (A, B) for 6 days. Treatment with different concentrations of metformin served as controls (C, D). After 6 days, prostasphere numbers were counted under the microscope and the proportion of sphere-generating cells was calculated by dividing the number of cells seeded by the number of prostaspheres (A, C). Prostaspheres from C4-2B cells treated with B-DIM (B) or metformin (D) were photographed and the results showed that 10 and 25 µM of B-DIM significantly reduced size and numbers of prostaspheres. n = 6, * P<0.05, ** P<0.01.

Tumor stem cells have the characteristics of forming tumorspheres**.** It has been shown that metformin could inhibit cancer stem cell growth, associated with AMPK activation [16]. In order to test whether B-DIM, which functions as an AMPK activator (Fig. 1, 2, 3) could target cancer stem-like cells and inhibit prostasphere formation, C4-2B cells were treated with different concentrations of B-DIM for 6 days in ultra-low adherent wells of 6-well plates. Treatment with metformin served as a control. The results showed that B-DIM inhibited prostasphere formation by 29% and 90% at treatment concentrations of 10 µM and 25 µM, respectively (Fig. 4), which is consistent with our previous findings [27]. The data demonstrate that B-DIM may possess the ability to suppress tumor stem-like cells in prostate cancer through activation of the AMPK pathway.

### B-DIM Activates AMPK and Down-regulates AR in Androgen Independent C4-2B Prostate Tumor Xenografts in SCID Mice

**Figure 5:**
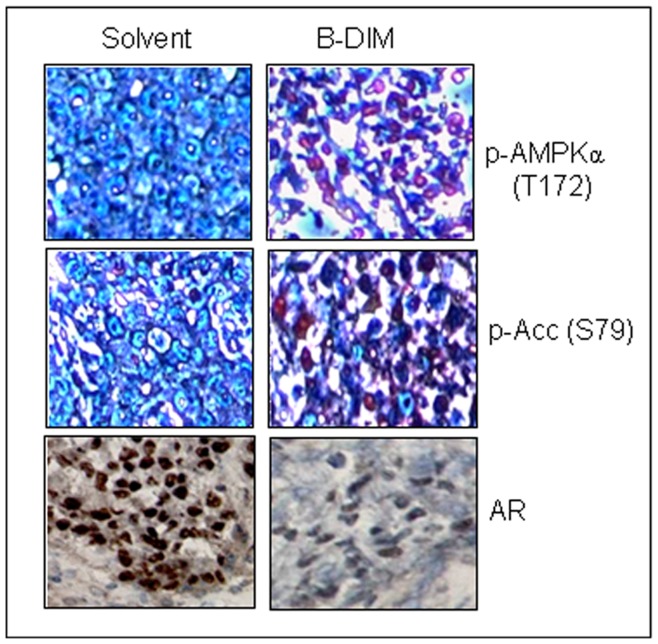
B-DIM activates AMPK signaling and inhibits AR expression in prostate tumor xenograft tissues. ICR SCID mice were implanted with C4-2B cells and treated with 5 mg/mouse of B-DIM by oral gavages daily for 4 wks. Tumor tissues were analyzed by immunohistochemistry using anti-phosphor-AMPKα (T172), phosphor-ACC (S79) or AR antibodies. The stained sections were visualized under the microscope (400×amplification). Unlike solvent-treated control, mice treated with B-DIM presented with activation of AMPK and significant loss of AR in tumor sections.

The above results from our *in vitro* studies clearly showed that the AMPK signaling pathway is one of the novel molecular targets of B-DIM in prostate cancer cells. To confirm this finding *in vivo*, we designed and used the experimental bone metastasis animal model (see Materials and Methods) that mimics bone metastasis of human prostate cancer. We found that B-DIM treatment *in vivo* inhibited C4-2B tumor growth within the bone microenvironment to some extent (20%; data not shown). The tumor tissues were removed and analyzed by immunohistochemistry using anti-phosphor-AMPKα, phosphor-ACC and AR antibodies. The results showed that p-AMPK- and p-ACC-positive cell populations increased significantly, while AR-positive cells decreased greatly in the tumors treated with B-DIM compared to the control (Fig. 5). These findings confirm that B-DIM is able to activate the AMPK pathway *in vivo*, associated with its anti-cancer activity.

## Discussion

The search for new antitumor drugs from natural sources is one of the most promising approaches for cancer prevention and therapy. We have been studying formulated 3,3′-Diindolylmethane (B-DIM) and showed that B-DIM can induce apoptosis and inhibit cell growth, angiogenesis, and invasion of prostate cancer cells by regulating NF-κB, Akt and AR signaling pathways [24]–[25]. However, the precise molecular mechanism(s) by which B-DIM elicit its anti-cancer effects on human prostate cancer have not been fully elucidated. In this study, we discovered that AMPK is one of the direct molecular targets of B-DIM in prostate cancer cells *in vitro* and *in vivo*.

The AMPK signaling pathway has recently become an important focus of interest in cancer prevention and therapy. Bowker *et al*. investigated the incidence of cancer in 10,309 diabetic patients treated with insulin, metformin or sulfonylureas for a period of 5 years. They reported that patients treated with metformin had a significantly lower rate of cancer-related mortality compared with patients exposed to other anti-diabetic medicines [12]. The major anti-cancer mechanism of metformin was associated with activation of AMPK signaling [34]. Activation of AMPK inhibits energy consuming pathways, protein synthesis and cell proliferation, through suppression of the mTOR pathway *via* tuberous sclerosis 2 protein (TSC-2) [38]. Hirsch H *et al*. reported that metformin selectively targets cancer stem cells and inhibits tumor growth in mouse models mediated by activation of the AMPK pathway [16]. It has been reported that some natural compounds including genistein (rich in soy bean), EGCG (abundant in green tea), and capsaicin (from hot pepper) are able to activate the AMPK pathway [17]. Discovery of more AMPK activators from natural sources is becoming an attractive approach to cancer prevention and therapy.

**Figure 6:**
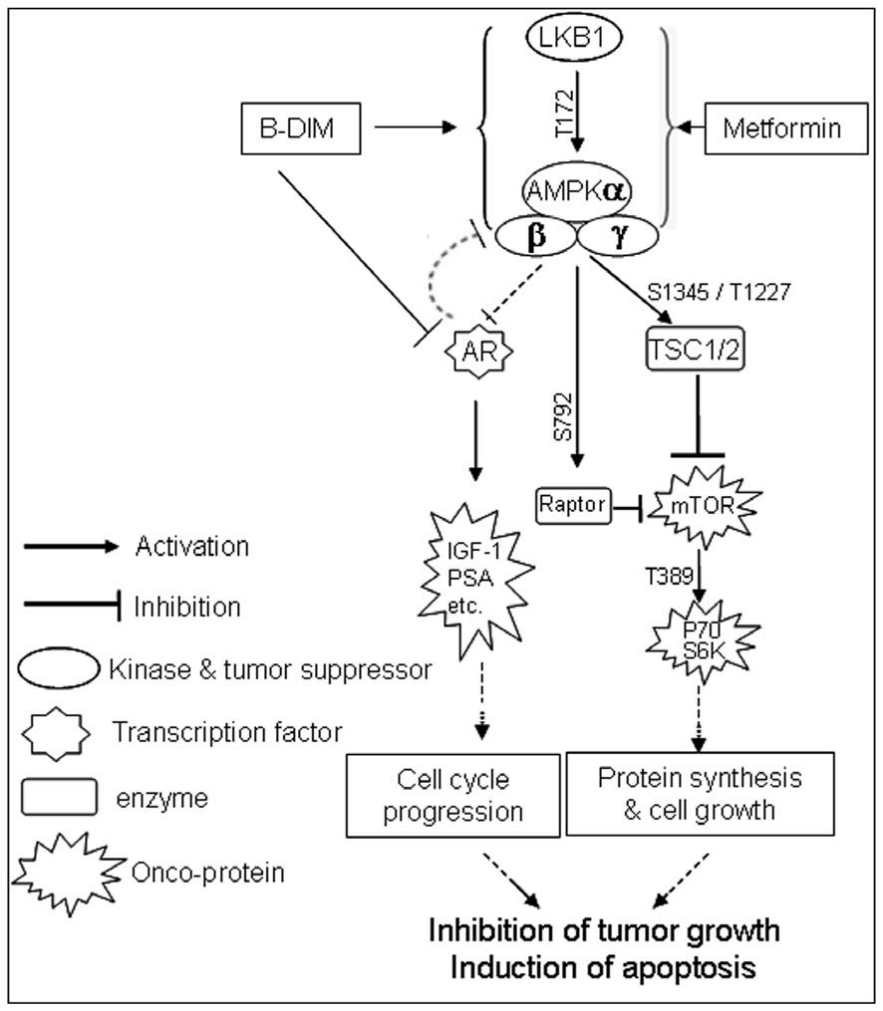
Schematic diagram showing the various mechanisms associated with inhibition of tumor growth and induction of apoptosis by B-DIM.

Our current findings show that B-DIM can activate AMPK signaling as early as three hours in both androgen-sensitive LNCaP and androgen-insensitive C4-2B prostate cancer cells, measured by: (i) increased protein levels of phosphor-AMPKα (T172), (ii) increased levels of phosphorylated ACC on serine residue 79 [30]–[31], (iii) increased protein levels of phosphor-Raptor (S792), which is a direct target of AMPK and an mTOR binding partner and inhibitor [8], and (iv) decreased levels of phosphor-mTOR (Fig. 1). Consequences of AMPK activation by B-DIM appear to be mediated through inhibition of AR and PSA expression, leading to the induction of apoptosis upon further treatment for an additional 21 hours (Figs. 1, 6). AMPK activation by B-DIM could be blocked by pre-treatment with Compound C, an AMPK inhibitor, in the prostate cancer cells (Fig. 2).

The androgen receptor (AR) is a critical factor for prostate cancer development and progression. Prostate cancer is dependent on androgen stimulation mediated by AR, and AR even plays an important role in cancer development and drug-resistance in androgen-independent prostate cancer cells. Alternative mechanisms of AR activation in androgen-independent prostate cancer are proposed through other signaling pathways, including ERKs, Akt, β-catenin and caveolin [39]–[41]. Therefore suppression of AR is one of the therapeutic strategies for prostate cancer patients. Casodex is an anti-androgen drug clinically used for patients in metastatic/advanced stage. Any natural compounds that could enhance the efficacy of casodex have potential therapeutic benefit in the clinic. We showed in the present study that co-treatment of cells with casodex and B-DIM led to a significant increase in phosphor-AMPKα and suppressed AR expression in prostate cancer cells (Fig. 3), demonstrating a novel mechanism for synergy of anti-AR therapy.

Prostate cancer tissue contains a rare population of multi-potent cancer stem cells with the capacity to self-renew. These prostate cancer stem cells could be enriched and measured by a colony formation assay in three-dimensional cultures, referred to as prostasphere formation. Prostate cancer cells with stem cell characteristics possess the ability to form prostaspheres from single cells as a condition for self-renewing in non-adherent culture conditions [42]. To determine if B-DIM could target prostate stem/stem-like cells, we tested it in prostasphere formation assays. The result showed that after six-day incubation of C4-2B cells with different doses of B-DIM, not only were numbers of formed prostaspheres significantly decreased, but the size of prostaspheres was reduced as well (Fig. 4). The findings from the *in vitro* studies were further confirmed by the immunohistochemical results from tumor tissues of prostate cancer xenografts treated with B-DIM (Fig. 5). Positive cell populations stained with phosphor-AMPK or phosphor-ACC were significantly increased, while AR-positive cells were reduced in the B-DIM treated tumor tissue (Fig. 5).

In summary, the present study provides the first evidence that the AMPK signaling pathway is one of the molecular targets of B-DIM for its anti-cancer activity. Activation of AMPK by B-DIM results in the suppression of its downstream target mTOR, down-regulation of AR expression and induction of apoptosis in both androgen-sensitive LNCaP and androgen-insensitive C4-2B prostate cancer cells (Fig. 6). Activation of AMPK by B-DIM was also observed in treated prostate tumors. Our findings demonstrate that B-DIM could be used as a potential agent in the clinic for the prevention and/or treatment of prostate cancer regardless of androgen responsiveness of the cells.
